# Identification and Quantification of Organic Contaminants and Evaluation of Their Effects on Amine Foaming in the Natural Gas Sweetening Industry

**DOI:** 10.1021/acsomega.2c05132

**Published:** 2022-12-08

**Authors:** Qingmei Chen, Xiujun Peng, Jingwen Xue, Jinjin Li, Chunfeng Pan

**Affiliations:** † Research Institute of Natural Gas Technology, PetroChina Southwest Oil & Gasfield Company, Chengdu610213, China; § National Energy R&D Center of High Sulfur Gas Exploitation, Chengdu610213, China; ∥ High Sulfur Gas Exploitation Pilot Test Center, CNPC, Chengdu610213, China; ‡ PetroChina Southwest Oil & Gasfield Company, Chengdu610213, China

## Abstract

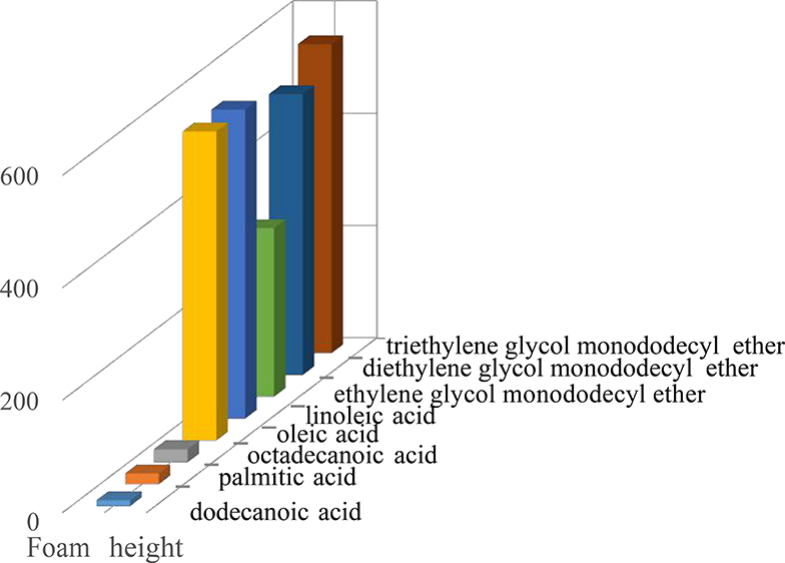

Contamination is a leading cause of corrosion, foaming, and amine-absorption capacity limitation, predominantly foaming. There is currently an urgent need to identify the sources of amine foaming and eliminate them or reduce their impacts. Gas chromatography–mass spectrometry (GC-MS) and a sample pretreatment method were developed to identify and quantify the organic contaminants. Linear hydrocarbons (C_12_–C_22_), long-chain carboxylic acids and esters, alcohol ethoxylates, and benzene derivatives were detected, characterized, and quantified in amine solutions. Furthermore, the effects of the contaminant concentrations on foaming behavior were also investigated by adding those contaminants. The results reveal that the main issue of foaming is due to the presence of unsaturated fatty acids and alcohol ethoxylates, even with a small amount of 10 ppm, whereas benzene derivatives like methylpyridine, quinoline, methyl naphthalene, benzyl alcohol, octahydroacridine, and linear hydrocarbons have little effect on amine foaming, even with an amount up to 2000 ppm. Therefore, it is necessary to monitor the existence and content of these surface-active contaminants.

## 1 Introduction

Methyldiethanolamine (MDEA) or MDEA-based solvents are widely used to remove hydrogen sulfide (H_2_S), carbon dioxide (CO_2_), and mercaptans (RSH) from sour feed gas.^1−5^ However, amine-solvent foaming is a tricky technical issue often encountered in the sour gas sweetening industry.^6−8^ In 2019, two amine foaming incidents, which occurred in the Anyue and Jian’ge natural gas plants in China, caused a number of severe impacts on the integrity of gas sweetening facilities, such as loss of solvent, reduction of vapor–liquid contact area, reduction of sales and gas supply, off-specification of sweet gas, fluctuation of operating parameters, and hence high revenue losses. Foaming can be attributed to numerous foam-inducing contaminants either brought into the system through the feed gas or generated inside the system, such as amine degradation and corrosion products.^9−11^

Considerable work has been carried out to study the effects of contaminants (e.g., liquid hydrocarbons, carboxylic acids, corrosion inhibitors, fine particulates, amine degradation products, and process parameters) on the foaming behavior and solution physical properties (e.g., viscosity, density, and surface tension)^12−16^ as well as defoaming means.^17−21^ However, in fact, more effort should be made to identify the sources of foam and reduce their impact or further eliminate them. Unfortunately, due to the complexity of the contaminants and the interference of the MDEA matrix, the isolation, identification, and quantification of the organic contaminants in amine solution remain a challenge. In addition, the concentrations of all compounds were reported as relative wt % in the literature rather than the absolute value of concentration. Therefore, there is currently an urgent need to identify and quantify the major sources of foaming for further foam control.

In the present work, a GC-MS analysis method was established to identify and quantify the main organic contaminants and then evaluate their effect on amine foaming by adding these compounds to fresh 40 wt % aqueous MDEA solution. The aim of this work is to find out the main sources that may be the predominant causes of amine foaming. The knowledge obtained from this work will be useful for the technology progress of contaminant removal.

## 2 Experimental Section

### 2.1 Materials

Fresh MDEA and lean MDEA were obtained from the natural gas purification plant of Jian’ge (Jiangyou, China) and from the natural gas purification plant of Yilong (Suining, China), respectively. *n*-Dodecane, *n*-tetradecane, *n*-hexadecane, *n*-octadecane, *n*-eicosane, and *n*-docosane were supplied from Macklin. Dodecanoic acid, palmitic acid, octadecanoic acid, oleic acid, linoleic acid, methyl palmitate, methyl oleate, ethylene glycol monododecyl ether, diethylene glycol monododecyl ether, and triethylene glycol monododecyl ether were purchased from Macklin. Methylpyridine, methylnaphthalene, octahydroacridine, and quinoline were obtained from Aladdin. Dichloromethane and ethanol were purchased from the Kelong Chemical Reagent Company (Chengdu, China). All chemicals used were of AR grade (≥99.5% purity) except for MDEA industrial samples. The purity of helium used as the carrier gas is required to be above 99.999%. Industrial grade nitrogen (N_2_) was purchased from a gas supplier (Chengdu, China). A stopwatch was used to record the bubbling time.

### 2.2 Instrumentation and Apparatus

GC-MS analysis was carried out on an Agilent 7890A-5977B GC-MSD instrument with an HP-INNOWAX MS capillary column (60 m × 0.25 mm i.d. × 0.25 μm film thickness).

GC conditions: the temperature program of the GC oven started at 110 °C, increased at 2.5 °C/min to 130 °C, was held for 5 min, and then increased to 180 °C at 5 °C/min with a hold time of 1 min and finally increased at 20 °C/min to 230 °C and held for 60 min. The temperature of the injector was set at 360 °C, and 2 μL of the sample was injected in split mode (split ratio 10:1). Helium was used as a carrier gas with a constant flow rate of 1 mL/min.

MS parameters: an electron impact (EI) resource was used in positive-ion mode with an EI energy of 70 eV and a mass range of 30–500 *m*/*z* in full-scan mode. The temperature of the ion source, four-stage rod, and the transfer line were set at 230, 150, and 290 °C, respectively. The solvent delay time was set at 5.5 min and the gain factor set at 1. Data were acquired and processed using Agilent Masshant software, and the NIST 17 mass spectrum library was used for the identification of relative compounds.

### 2.3 Standard Solution Preparation

A stock solution (500 μg/mL) of a standard mixture was prepared by dissolving the accurate amount of the standard compounds in ethanol. The standard working solution was obtained by further dilution of stock solution with ethanol. Nine calibration levels of mixed standards with a concentration of 0.01, 0.1, 1, 10, 20, 30, 40, 50, and 100 μg/mL were prepared to investigate the linearity.

### 2.4 Sample Pretreatment

An amount of 25 g of the amine sample was weighted into a 100 mL beaker, and 1:1 HCl (v/v) was added to neutralize MDEA. The pH of the solution was adjusted to around 2, and then it was transferred to a 250 mL separatory funnel. Subsequently, the beaker was rinsed with 5 mL of dichloromethane (CH_2_Cl_2_) three times, and we transferred them to the separating funnel. Finally, the mixture was partitioned with 20 mL of CH_2_Cl_2_ for about 5 min. After the partition, the lower CH_2_Cl_2_ phase was collected into a weighing bottle and concentrated to 250 μL of a sample bottle for GC-MS testing.

### 2.5 Validation Study

The mixed standard was used for validation, and the parameters like linearity and limit of detection (LOD) were evaluated during the validation of the analytic method. Nine mixed standards were prepared for calibration levels ranging from low to high over 10 000 times to study the linearity. The method’s LOQ was calculated based on the minimum amount of compound analyzed by GC-MS, and the signal-to-noise (S/N) ratio of the compound was 3. For most compounds, the relative standard deviation (RSD) is less than 20%.

In this study, full-scan mode was used for qualitative analysis, and the selective ion monitoring (SIM) mode was adopted for quantitative analysis. Quantitation was identified by using an external standard method with standard solution mixtures.

### 2.6 Foaming Experiments

#### 2.6.1 Foaming Experimental Setup

**Figure 1:**
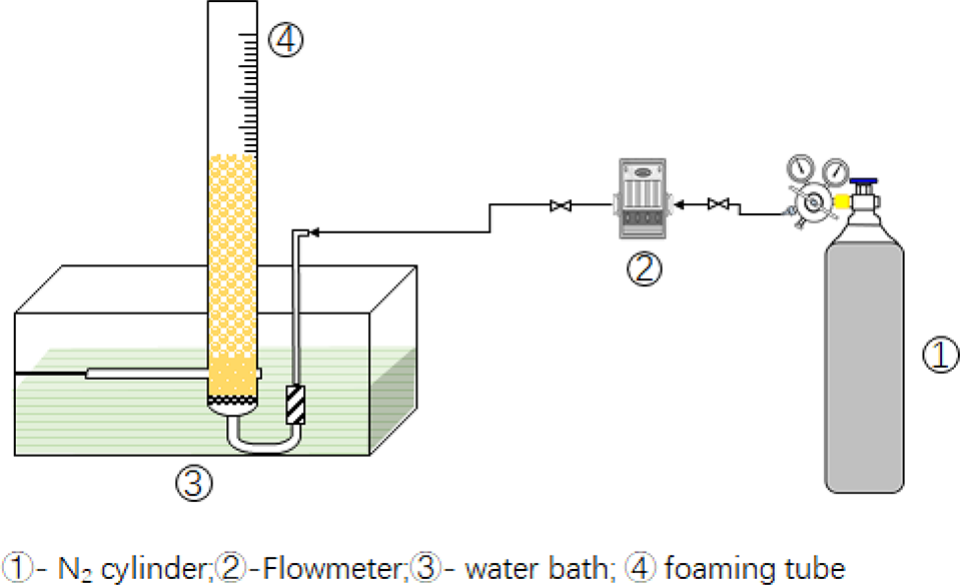
Schematic diagram of a foaming experimental device.

Based on the standard ASTM D892 test method for the foaming behavior of lubricating oils (ASTM D892, 1999), the experimental device consisted of a industrial-grade nitrogen source, a flow meter (0–1.0 L/min), a foaming tube with scale, and a No. 3 glass sand inserted into the foaming tube and consisted of a water bath with accuracy of ±0.1 °C, as shown in Figure 1. The foaming tube was usually a glass tube with an inner diameter of 30 mm and a height of 500 mm. The industrial-grade nitrogen (N_2_) was utilized as an inert gas, and No. 3 glass sand was used to produce dispersed gas for the foam test.

#### 2.6.2 Foaming Experimental Procedures

The test solution was prepared by adding each contaminant to 40 wt % MDEA solution in parts per million (ppm) concentrations. Prior to each experiment, the foaming tube containing 100 mL of tested solution was placed in a water bath and heated to 40 °C for approximately 10 min to reach thermal equilibrium, and then the nitrogen valve was opened. The nitrogen at a constant flow rate of 0.25 L/min entered the bottom of the foaming tube and passed through the No. 3 glass sand to produce bubbles in MDEA solution. The foam height generated at the top of the liquid level was recorded after a blowing time of 5 min. The foaming break time was the time resistance of the last bubble to break into a continuous liquid phase. Prior to testing each contaminant, 40 wt % MDEA solution without any additive was used as a benchmark for the full-dose trials. The foaming tendency of MDEA was reported in terms of foam height. Foam stability was reported on the basis of break time. All the experiments were repeated thrice to obtain the standard deviations below 5%.

## 3 Results and Discussion

### 3.1 Validation Study

GC-MS technology combines the advantages of high-efficiency separation by chromatography and qualitative analysis by mass spectrometry, so it has become one of the most favorable means for analyzing mixtures. Because isomeric forms of linear hydrocarbons, alcohol ethoxylates, and long-chain carboxylic acids usually present quite similar mass spectra, retention time remains the decisive criterion for identification of a substance. To guarantee the correct identification of analytes in real amine samples, the studied compounds were verified by comparison of retention times and MS spectra with that of standard.

**Figure 2:**
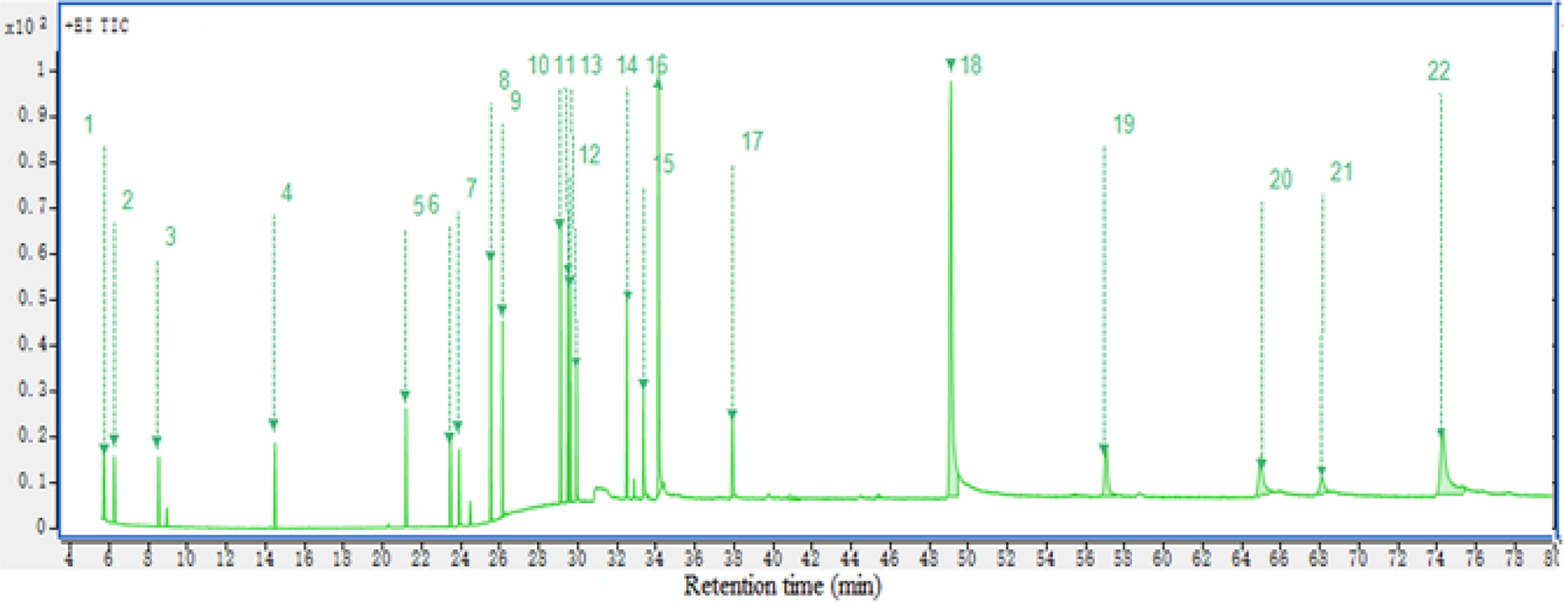
Total ion chromatogram (TIC) of standard solution with 50 mg/L of each compound.

Under the optimal GC-MS conditions, a standard sample containing 22 compounds was completely separated and identified, and the total ion chromatogram of the standard sample was shown in Figure 2. The separated compounds were quantified in SIM mode. For each target substance, the most intensive characteristic ions were chosen. The identified compounds and their retention times, the characteristic selected ions, the parameters of the calibration curves, and the LOD values are listed in Table 1.

**Table 1:** Retention and Statistical Characteristics of Tested Analytes^a^

compound	peak mark	Rt/min	selected ion (*m*/*z*)	*r*^2^	linear range	LOD (mg/L)
*n*-dodecane	1	5.75	43.1, 57.1, 71.1, 85.1	0.999	0.01–100	0.01
methylpyridine	2	6.29	51.0, 66.1, 78.0, 93.1	0.999	0.01–100	0.01
*n*-tetradecane	3	8.55	43.1, 57.1, 71.1, 85.1	0.999	0.01–100	0.01
*n*-hexadecane	4	14.50	43.1, 57.1, 71.1, 85.1	0.999	0.01–100	0.01
*n*-octadecane	5	21.21	43.1, 57.1, 71.1, 85.1	0.999	0.01–100	1
methylnaphthalene	6	23.50	115.0, 141.1, 142.1	0.998	0.01–100	0.01
benzyl alcohol	7	23.9	77.0, 79.0, 107.0, 108.0	0.999	0.01–100	0.01
quinoline	8	25.57	102.0, 103.0, 128.1, 129.0	0.999	0.01–100	0.01
*n*-eicosane	9	26.14	43.1, 57.1, 71.1, 85.1	0.999	5–100	5
*n*-docosane	10	29.10	43.1, 57.1, 71.1, 85.1	0.999	5–100	5
methyl palmitate	11	29.49	74.0, 87.0, 143.1, 227.2	0.997	5–100	5
*n*-phenylcyclohexylamine	12	29.64	132.1, 175.1, 118.1	0.998	0.01–100	0.01
ethylene glycol monododecyl ether	13	29.91	43.1, 57.1, 71.1, 85.1, 97.1	0.998	1–100	1
octahydroacridine	14	32.53	186.1, 159.1, 172.1	0.998	0.01–100	0.01
9-methyl oleate	15	33.35	55.1, 69.1, 83.1, 97.1, 264.2	0.998	5–100	5
dodecanoic acid	16	34.14	60.0, 73.0, 129.0, 157.1	0.999	1–100	1
diethylene glycol monododecyl ether	17	37.94	45.0, 57.1, 71.1, 85.1	0.998	1–100	1
palmitic acid	18	49.12	60.0, 73.0, 129.0, 213.2	0.998	5–100	5
triethylene glycol monododecyl ether	19	57.02	45.1, 57.1, 71.1, 89.1	0.998	10–100	10
octadecanoic acid	20	64.94	45.1, 60.0, 73.0, 129.1	0.999	5–100	5
oleic acid	21	68.14	55.0, 69.0, 73.0, 97.1	0.998	10–100	10
linoleic acid	22	74.10	45.1, 55.1, 67.0, 81.1, 95.1	0.998	10–100	10

a Rt: retention time. *r*^2^: linear regression data. LOD: limit of detection.

Calibration curves for most analytes were linear with *r*^2^ values greater than 0.999. For polar substances like alcohol ethoxylates and long-chain carboxylic acids, the curves were more likely to not include some of the lower calibration levels due to sensitivity issues. However, since nine calibration standards were used to construct the calibration curves, at least 5–6 data points could still generate curves, if a few lower calibration levels were not used due to poor data quality.

As shown in Figure 2, weakly polar substances like linear hydrocarbons (C_12_∼C_22_), methylpyridine, methylnaphthalene, 2-butoxyethanol, and benzyl alcohol were first eluted, and then the medium-polar substances like higher fatty acid esters were eluted. Finally, the polar substances like alcohol ethoxylates and long-chain carboxylic acids were eluted. Figure 2 clearly shows the complete separation of all tested analytes. The response intensity of weakly polar substances was significantly higher than that of polar substances under the same concentration of 50 mg/L. This difference could be caused by the different efficiency of ionization of these compounds and the low intensity of characteristic ions selected for quantification purposes. Therefore, it is necessary to use a calibration standard instead of the area normalization method for quantitative analysis.

### 3.2 Application to Sample Analysis

**Figure 3:**
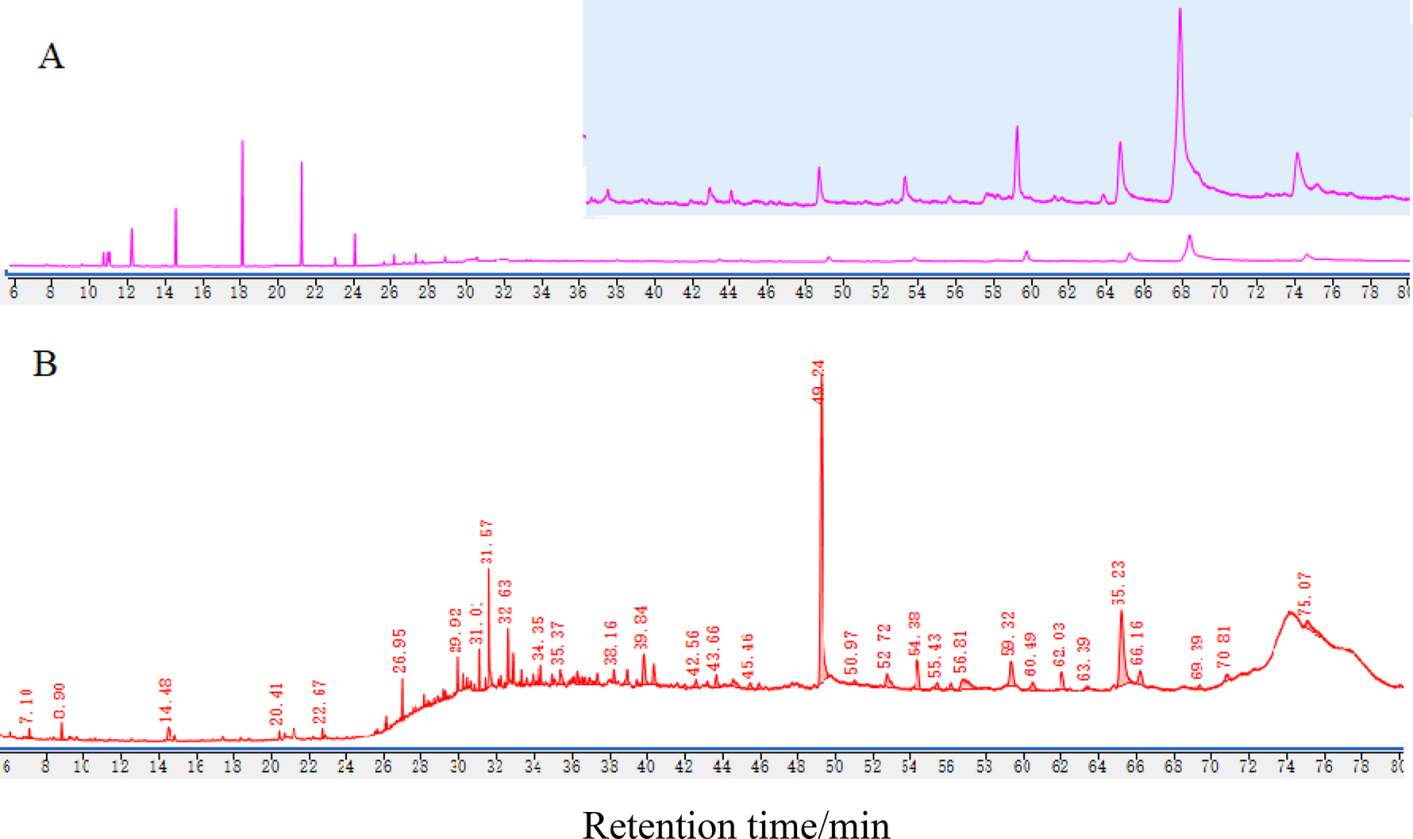
Chromatogram (TIC) of samples A (Jian’ge natural gas plant) and B (Yilong natural gas plant).

Industrial lean amine samples with severe foam were collected from two natural gas purification plants in Jian’ge and Yilong, which were then extracted and analyzed by GC-MS. Based on the mass spectral information and retention time of each compound, the corresponding peaks were identified. Figure 3 shows the total ion chromatograms (TIC) of amine samples. The quantitative analysis was carried out by eq 1 below, and the results of each component are listed in Table 2.
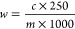
1where *w* is the mass concentration in μg/g; *c* is the concentration calculated from the standard curve in μg/mL; and *m* is the weighted mass of sample in g.

**Table 2:** Absolute Quantitative Analysis of Samples^a^

		content (μg/g)	
no.	compound	sample A	sample B
1	*n*-dodecane	0.06	0.58
2	methylpyridine	—	—
3	*n*-tetradecane	0.34	—
4	*n*-hexadecane	0.24	—
5	*n*-octadecane	0.46	0.02
6	methylnaphthalene	—	—
7	benzyl alcohol	—	—
8	quinoline	—	—
9	*n*-eicosane	0.08	0.03
10	*n*-docosane	0.08	0.02
11	methyl palmitate	—	0.02
12	*n*-phenylcyclohexylamine	—	0.005
13	ethylene glycol monododecyl ether		1.2
14	octahydroacridine	—	—
15	9-methyl oleate	0.3	0.09
16	dodecanoic acid	0.3	0.16
17	diethylene glycol monododecyl ether	9.45	0.18
18	palmitic acid	6.81	5.03
19	triethylene glycol monododecyl ether	0.537	0.30
20	octadecanoic acid	25.47	10.42
21	oleic acid	0.67	1.73
22	linoleic acid	15.0	43

a “—” not detected.

As shown in Figure 3 and Table 2, although the response intensity of linear hydrocarbons was obvious in the TIC chromatogram, the content in the sample was actually very low without 1 μg/g (1 ppm). Alcohol ethoxylates and long-chain carboxylic acids (palmitic acid, octadecanoic acid, and oleic acid) were found in real samples, although their response intensity was low. The oleic acid was found with the highest content in sample A, which was 30 μg/g. Octadecanoic acid was 25.47 μg/g; palmitic acid was 6.81 μg/g; and diethylene glycol monododecyl ether was 9.45 μg/g. The octadecanoic acid was found with the highest content in sample B, which was 10.42 μg/g, and palmitic acid was 6.03 μg/g. These compounds that were detected in this study are usually present in other gas sweetening solvents.

### 3.3 Effects of Different Contaminants on Foam Formation

In order to reveal the effects of contaminants on aqueous 40 wt % MDEA foaming behavior and identify the main sources of foaming, different additives were added to aqueous 40 wt % MDEA solution to conduct foaming experiments. Table 3 shows their effects on the foam height and break time. Clearly, fresh 40 wt % MDEA solution can not form stable foam generally, while the fatty acid esters like methyl palmitate and 9-methyl oleate and alkanes represented by C_22_ alkanes and benzene derivatives (e.g., methylpyridine, methyl naphthalene, and quinoline) have been shown to be ineffective on amine foaming, even with an enormous amount up to 2000 ppm.

**Table 3:** Effect of Additives on Foaming Behavior for 40% MDEA Solution

additive	concentration of additive/ppm	foaming height/mm	break time/s
fresh 40 wt % MDEA	0	5	3
*n*-C_22_H_46_	2000	20	5
methyl palmitate	2000	10	3
9-methyl oleate	2000	15	3
quinoline	2000	20	12
methylpyridine	2000	5	1
methylnaphthalene	2000	5	1

**Figure 4:**
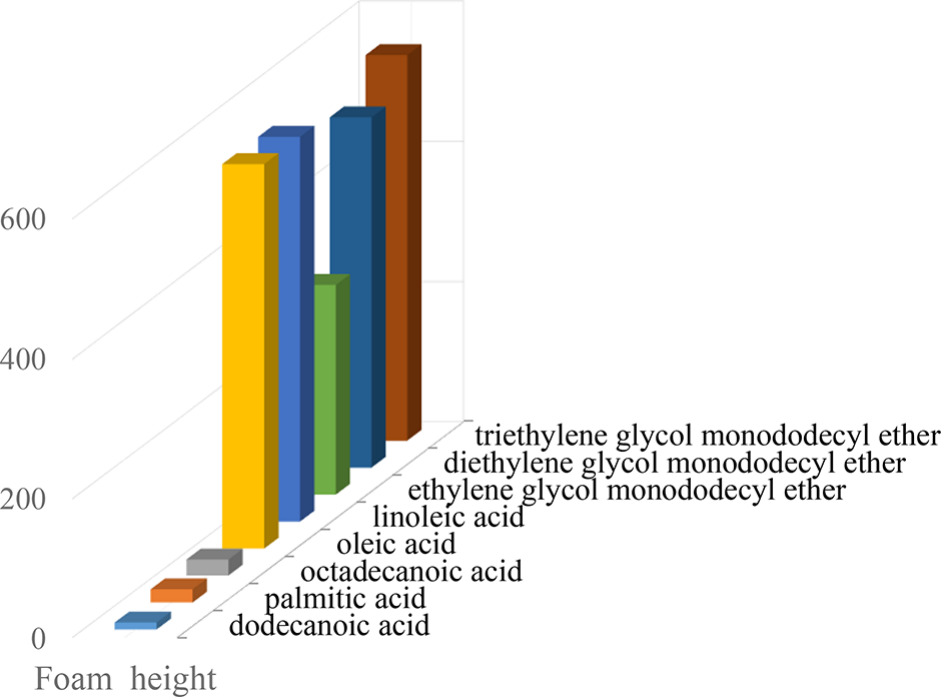
Effect of 10 ppm higher fatty acid addition on foam height.

**Figure 5:**
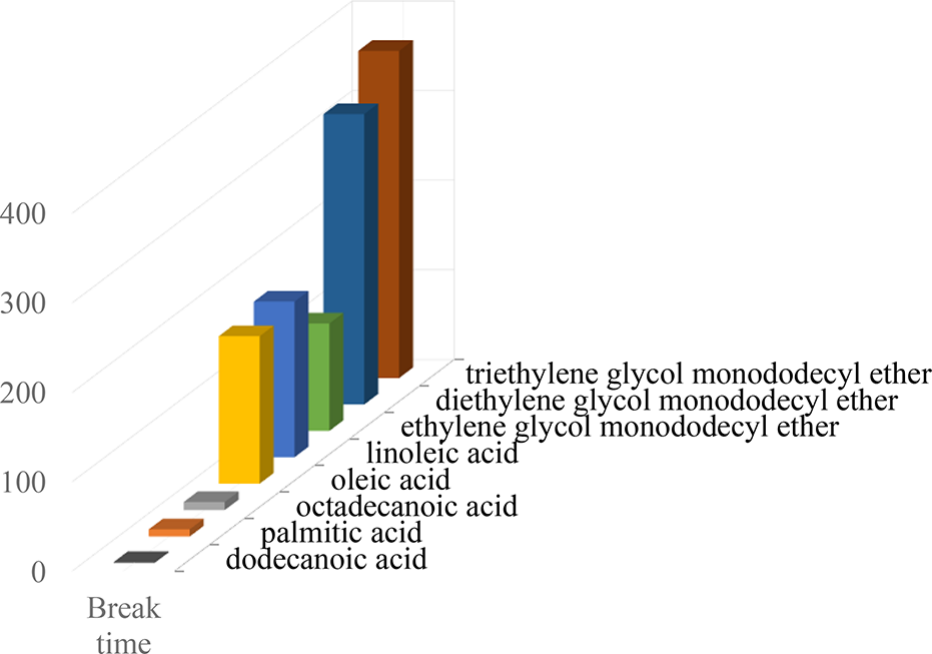
Effect of 10 ppm higher fatty acid addition on break time.

The effect of long-chain carboxylic acids and alcohol ethoxylates was studied by adding the five long-chain carboxylic acids and three alcohol ethoxylates with a concentration of 10 ppm into aqueous 40 wt % MDEA solution. The results in Figure 4 and Figure 5 clearly show that alcohol ethoxylates and unsaturated fatty acids with a concentration of 10 ppm can significantly increase foam tendency and foam stability. Among them, triethylene glycol monododecyl ether, diethylene glycol monododecyl ether, and ethylene glycol monododecyl ether, as well as linoleic acid and oleic acid, have the greatest effect on 40 wt % MDEA foaming behavior, whereas saturated fatty acids (e.g., dodecanoic acid, palmitic acid, and octadecanoic acid) with a concentration of 10 ppm have no apparent effect.

**Figure 6:**
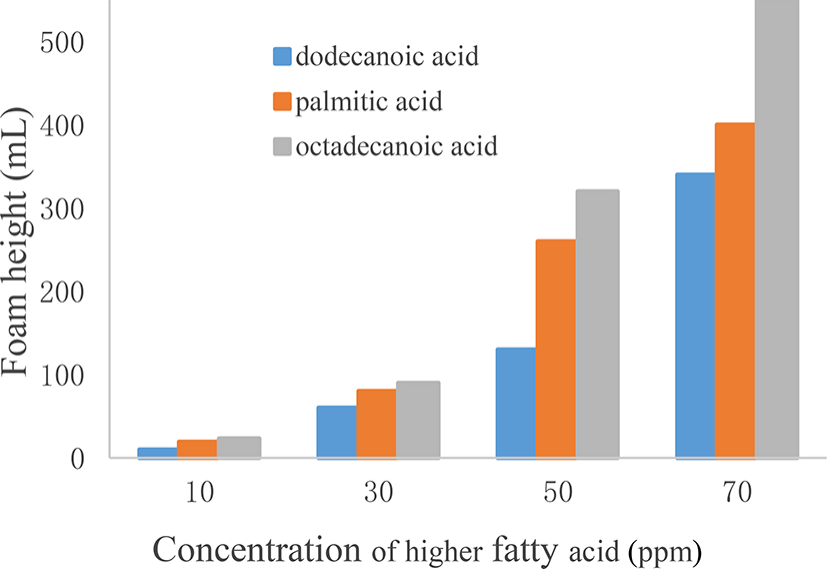
Effect of higher fatty acid addition on foam height.

**Figure 7:**
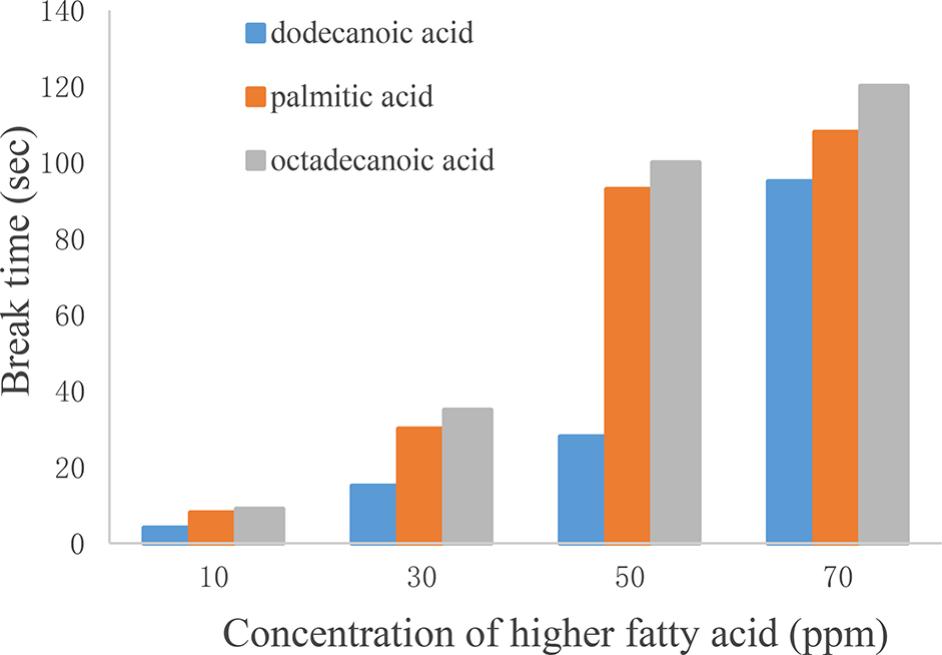
Effect of higher fatty acid addition on break time.

Also the effect of concentrations with saturated fatty acids (e.g., dodecanoic acid, palmitic acid, and octadecanoic acid) was tested by varying the concentration from 10 to 70 ppm. As shown in Figure 6 and Figure 7, with the increase of saturated fatty acid concentration, the foam performance showed an ascent trend by various degrees. The greater the concentration was, the more obvious the foam behavior. Once the concentration increased to more than 30 ppm, foams were produced, and the foam tendency and foam stability further increased with concentration. In addition, at the same concentration, the foamability of long-chain fatty acids was stronger than that of short-chain fatty acids.

The above results illustrate that alcohol ethoxylates and all long-chain carboxylic acids, especially unsaturated fatty acids, have a distinct effect on aqueous 40 wt % MDEA solution. This is consistent with the fact that alcohol ethoxylates and long-chain carboxylic acids are a nonionic surfactant and anionic surfactant, which typically consist of a polar group and a nonpolar group (a long alkyl chain). These two surfactants can form strong interactions with amine solution and thereby decrease the interfacial tension. The observations imply that these two surfactants might be the major foam promoters and play a critical role in amine foaming. The order of effect with regard to foam tendency and foam stability is triethylene glycol monododecyl ether > diethylene glycol monododecyl ether > linoleic acid > oleic acid > ethylene glycol monododecyl ether ≫ octadecanoic acid > palmitic acid > dodecanoic acid.

The benefit of this paper is to identify and confirm the sources of amine foaming. The highest contributors to the foaming behavior are long-chain carboxylic acids and alcohol ethoxylates, specifically oleic acid, linoleic acid, diethylene glycol monododecyl ether, and triethylene glycol monododecyl ether. These findings well answer the reasons for the serious foaming of sample A and sample B, in which the oleic acid, linoleic acid, diethylene glycol monododecyl ether, and triethylene glycol monododecyl ether were found and are the main sources of foaming. Thus, it is very necessary to monitor the contents of these contaminants and eliminate them to assure amine solvent quality.

## 4 Conclusion

A reliable gas chromatography–mass spectrometry (GC-MS) method, together with the process of sample pretreatment, were developed to identify and quantify the contaminants in amine solution. Linear hydrocarbons (C_12_∼C_22_), long-chain carboxylic acids and fatty acid esters, alcohol ethoxylates, and benzene derivatives can be well separated and detected. The main advantages of this method are the direct analysis of long-chain carboxylic acids without the derivatization process. Meanwhile, the effects of these contaminants have been discussed by addition of different concentrations of contaminants on 40 wt % MDEA solution. The results reveal that most influential foam-inducing contaminants are alcohol ethoxylates and unsaturated fatty acids. They are triethylene glycol monododecyl ether, diethylene glycol monododecyl ether, ethylene glycol monododecyl ether, linoleic acid, and oleic acid, which have been proven to play a key role in the foam tendency and stability, even with a small amount of 10 ppm. Thus, the best approach to abate foaming is to prevent the ingress of contaminants in feed gas by adding antifoam agents at the wellhead or by using effective filter separators. Once the contaminants have invaded the amine unit, the amine liquid reactivation device, where a special designed adsorbent is filled in the absorption tank, has proven to be effective in removing contaminants from amine solution.
